# Molecular Determinants of the Spacing Effect

**DOI:** 10.1155/2012/581291

**Published:** 2012-03-22

**Authors:** Faisal Naqib, Wayne S. Sossin, Carole A. Farah

**Affiliations:** ^1^Department of Physiology, Montreal Neurological Institute, McGill University, Montreal, QC, Canada H3A 2B4; ^2^Department of Neurology and Neurosurgery, Montreal Neurological Institute, McGill University, Montreal, QC, Canada H3A 2B4

## Abstract

Long-term memory formation is sensitive to the pattern of training sessions. Training distributed over time (spaced training) is superior at generating long-term memories than training presented with little or no rest interval (massed training). This spacing effect was observed in a range of organisms from invertebrates to humans. In the present paper, we discuss the evidence supporting cyclic-AMP response element-binding protein 2 (CREB), a transcription factor, as being an important molecule mediating long-term memory formation after spaced training. We also review the main upstream proteins that regulate CREB in different model organisms. Those include the eukaryotic translation initiation factor (eIF2*α*), protein phosphatase I (PP1), mitogen-activated protein kinase (MAPK), and the protein tyrosine phosphatase corkscrew. Finally, we discuss PKC activation and protein synthesis and degradation as mechanisms by which neurons decode the spacing intervals.

## 1. Introduction

Memory retention is highly sensitive to the pattern of trials used during training. Spaced training, where training trials are presented with intervening rest periods, produces longer lasting memories than massed training, where an equal amount of trials are presented consecutively without rest periods [1–6]. This phenomenon is called the spacing effect and was first described in 1885 by the German psychologist Hermann Ebbinghaus [7]. More than a century later, the spacing effect is recognized as a pedagogical tool and receiving serious consideration at a number of levels [8–10].

 The spacing effect was observed in humans in a variety of tasks and contexts [1, 11, 12]. However, this effect is not limited to humans but has been reported in different species ranging from invertebrates to primates [11, 12]. In the present article, we review the current knowledge of the molecular basis of the effects of spaced training taking into consideration the different model organisms in which it has been studied. At the cellular level, spaced training is represented by synaptic plasticity generated from spaced stimulations. While certain forms of synaptic plasticity result from stimuli acting in the time scale of milliseconds, such as spike-timing-dependent plasticity [13–15], or seconds, such as coincidence detection [16], we focus in this paper on spacing periods on the order of minutes. While training trials separated by hours or days also lead to enhanced memories, the molecular mechanisms underlying these effects of spacing are likely to be distinct from those involved in distinguishing the shorter spacing intervals we focus on in this paper and thus will not be discussed.

## 2. Molecular Basis of the Spacing Effect

At the molecular level, the increased ability to generate long-term memories from spaced trials compared to massed trials should be reflected by the increased ability of spaced trials to activate molecular pathways important for memory formation. Multiple studies from different model organisms suggest that activation of the transcription factor cyclic AMP response element-binding protein (CREB) is involved in such a pathway. CREB is of particular interest since, as described below, stimuli that normally only lead to short-term synaptic changes and memories can also lead to long-term synaptic changes and long-term memories if CREB is activated. This makes CREB an attractive molecule which activation would enhance memories seen after spaced training. In the following section, we will review evidence suggesting an important role for CREB as a key molecular mediator of the enhancement seen after spaced training in *Aplysia californica*, *Drosophila melanogaster*, and rodents; three model organisms used extensively in the literature to dissect out the molecular components underlying learning and memory formation [17, 18]. We will also discuss a more direct mechanism by which neurons can detect a spacing interval on the order of minutes and how this may be relevant for activation of CREB. In particular, we will discuss our recent findings that synthesis of proteins with fast degradation rates may be a mechanism for neurons to decode spacing intervals on the order of minutes [19].

## 3. Role of CREB in Long-Term Memory Formation

Multiple studies from different model organisms suggest that the persistence of long-term memory at both the behavioral and cellular levels requires protein synthesis and gene expression. The activation of CREB seems to be a crucial step in the induction of gene expression that mediates the formation of long-lasting memories [17, 18, 20–25]. The first reports of CREB being critical for the synaptic plasticity underlying memory came from *Aplysia*. This marine mollusk offers the advantage of having a small number of neurons which are large and easily identifiable [23], making it possible to characterize the contribution of each individual neuron to a learned behavior. One simple behavior studied extensively in *Aplysia* is the gill withdrawal reflex. When a tactile stimulus is applied to the animal's siphon, a defensive withdrawal reflex consisting of withdrawal of the siphon and gill is elicited. If the tactile stimulus is repeatedly applied, the animal becomes habituated and weakens its response (in amplitude and duration) to the stimulus [26]. This reflex is also subjected to sensitization. The same tactile stimulus applied to the siphon will lead to an enhanced response if the animal previously encountered an aversive stimulus such as a shock to the tail. Changes in the strength of the synapses formed between mechanoreceptor sensory neurons and withdrawal motor neurons were shown to participate in the formation of habituation and sensitization memories in *Aplysia* [23]. The decrease in synaptic strength that underlies habituation is referred to as synaptic depression and the increase in synaptic strength that underlies sensitization is referred to as synaptic facilitation. The precise mechanisms underlying synaptic depression are still unclear but are thought to involve an activity-dependent switching off of neurotransmitter release sites that is initiated by Ca^2+^ influx during individual action potentials [27]. On the other hand, synaptic facilitation is mediated by 5HT that is released by interneurons which are activated by the noxious stimulus [28, 29]. As in other systems, long-term memory formation depends on the number of training trials and the spacing between the trials. Four to five sensitization trials delivered at 15 min intervals induce long-term memory lasting more than 24 hours, whereas four or five massed trials fail to induce long-term memory [5].

Another advantage of *Aplysia* is the ability to recapitulate synaptic plasticity underlying memory formation in a cultured preparation consisting of primary sensory neurons and motor neurons. In these cultures, the sensory and motor neurons recreate synaptic connections that can be modified by 5HT applications, similarly to what is seen in the intact animal [24]. In culture, spaced applications of 5HT are superior to massed applications at generating long-term facilitation (LTF) of sensory-motor neuron synapses [30], thus allowing for the study of the spacing effect in a reduced preparation. Pioneering studies in *Aplysia* showed that LTF generated by spaced training requires the activity of transcription factors belonging to the CREB family [31–34]. Moreover, not only is CREB required for long-term memory formation after spaced training, it is the rate-limiting step [35]. Thus, manipulating the CREB pathway such that a single stimulus or massed stimuli can activate CREB allows for long-term memory formation from these stimuli. For example, decreasing the levels of the CREB repressor in cultured *Aplysia* neurons results in LTF being formed from just one application of 5HT, whereas uninjected neurons require multiple spaced applications of 5HT [32] suggesting an important role for CREB in regulating the spacing effect.

 CREB activation has also been found to be involved in long-term memory formation in the fruit fly *Drosophila melanogaster*. In this system, Pavlovian olfactory learning is used to train flies to avoid an odor paired with an electrical shock [6, 36, 37]. Testing of memory retention is carried out by exposing the flies to the electric shock paired odor and a second unconditioned odor, and observing the flies preference. Consolidated memory formed after olfactory learning is composed of two genetically distinct components: anesthesia-resistant memory (ARM) and long-term memory (LTM) [6]. ARM decays to zero within 4 days after training and is insensitive to the protein synthesis inhibitor cycloheximide [6]. LTM shows no decay over at least 7 days, and its formation is cycloheximide sensitive. Massed training, consisting of ten consecutive training trials with no rest intervals, produces only ARM, whereas spaced training, where ten consecutive trials are presented with a 15 min intertrial interval, produces LTM [2, 4, 6]. Thus, in this system, the formation of a protein-synthesis-dependent long-term memory requires spaced training. The long-term memory formed by spaced training is specifically blocked by expression of the CREB repressor [38]. Spaced training, but not massed training, also produces molecular traces in the olfactory circuit that were shown to be CREB-dependent [39, 40]. These molecular traces were represented by increased calcium influx into the axons of gamma mushroom body neurons of *Drosophila* after spaced olfactory conditioning.

In rodents, the spacing effect has been studied using a number of different learning tasks. A detailed analysis of the different behavioral paradigms is beyond the scope of this paper and we will focus on a small number of tasks where similar spacing intervals have been examined, and where a role for CREB has been shown. In particular, we will examine aversive conditioning paradigms where spatial or auditory information is coupled to a foot shock, and the animal expresses memory by fear responses such as freezing when exposed to the conditioning cue. In addition, we will examine a nonaversive task: novel object recognition, where rodents demonstrate their recognition of a previously encountered object by spending more time examining the novel object. Both of these memories show the spacing effect. In both contextual and auditory fear conditioning, spaced intervals lead to stronger memories for fear (increased freezing) than do massed trials [3, 41]. In novel object recognition, increasing spacing from 5 min to 15 min markedly improves the discrimination ratio between the novel and previously seen object [42]. Spaced training is superior to massed training in activating CREB, both in fear conditioning and novel object recognition [3, 41, 42].

The first studies in which the CREB gene was disrupted in rodents came from Hummler and colleagues [43]. In mammals, CREB is a large family of transcription factors that includes many isoforms generated by alternative splicing. Hummler and coworkers generated CREB mice that lacked the two main CREB isoforms *α* and *δ* [43]. Bourtchuladze and colleagues showed that the CREB mice formed intact short-term memory (tested 30 min after training) in contextual fear conditioning, but the long-term memory tested 24 hr after training was impaired [44]. Kogan and colleagues later showed that additional spaced training can overcome the profound memory deficits of CREB mice [45]. Increasing the intertrial interval from 1 to 60 minutes overcame the memory deficits of animals in contextual fear conditioning and spatial learning. Thus, manipulations of CREB function can affect the number of trials and the intertrial interval required for committing information to long-term memory. These mutant mice were not devoid of all CREB protein; it is believed that they retain ~15% of CREB activity from other minor CREB isoforms and modulatory proteins [46, 47]. While it appears that some memories can be made in the absence of CREB in the hippocampus [48], there may be compensation in this case from other members of the CREB family [48]. There may also be an important role for CREB in the amygdala [49–51]. Finally, similar to *Aplysia*, manipulating CREB to make it activated more easily, for example, by overexpressing CREB, or inhibiting a CREB phosphatase, allows massed training to generate the long-term memory previously only seen with spaced training [3, 42].

At the cellular level, a promising candidate for a form of synaptic plasticity linked to memory is long-term potentiation (LTP) [52–54]. Brief high-frequency stimulation in many pathways of the rodent brain produce an increase in synaptic strength [53]. This activity-dependent increase in synaptic strength is called LTP. LTP has 2 phases: the early phase (E-LTP), which lasts 1–3 hours and does not require protein synthesis, and the late phase (L-LTP) that requires both protein synthesis and gene expression [20, 22, 25, 55]. E-LTP is largely mediated by increased trafficking of AMPA receptors, by regulation of phosphorylation of the GluA1 subunit, or by activation of protein kinases that regulate trafficking of receptors containing GluA1 subunits [14, 15]. In contrast, L-LTP appears to be mediated by translation of a constitutively active protein kinase, termed PKM*ζ* (see below). PKM*ζ* stabilizes AMPA receptors containing GluA2 [56]. The spacing effect has largely been studied in the context of late protein synthesis-dependent LTP. In hippocampal slices, applying 100 Hz tetraburst stimulation in the CA1 area results in LTP. However, stimulation with 5 min interburst intervals (spaced training) results in a greater protein synthesis-dependent L-LTP when compared to a 20 sec interburst interval (massed training) [41]. Consistent with these findings, the loss of CREB removes some forms of late-LTP [57], while other forms are spared.

## 4. Proteins Acting Upstream of CREB to Regulate the Spacing Effect

As mentioned above, CREB activity has been implicated as the switch to form long-term memories from spaced training, but how does spaced training specifically lead to CREB activation? Evidence for several upstream signaling molecules has been reported. One such molecule is the *α* subunit of the eukaryotic translation initiation factor 2 (eIF2*α*). Phosphorylation of eIF2*α* inhibits general translation but selectively stimulates translation of ATF4, a repressor of CREB-mediated L-LTP and long-term memory [32, 58]. Costa-Mattioli and colleagues reported that in eIF2*α* ^+/S51A^ mice, in which eIF2*α* phosphorylation is reduced, the threshold for eliciting L-LTP in hippocampal slices is lowered, and spatial learning is enhanced in contextual fear conditioning [59]. Phosphorylation of eIF2*α* is normally reduced during spaced learning, and blocking this reduction blocks learning [59]. It will be interesting to determine if this depends on spacing, and if so how spacing is important for this regulation of eIF2*α* phosphorylation. Another repressor of CREB activation is protein phosphatase I (PP1), which reduces CREB activity by reversing activation of CREB by kinases [60]. Genoux and colleagues generated transgenic mice in which PP1 activity was reduced [42] and tested the effect on the animal's performance in the object recognition task. In this task, memory for objects is inferred from the animal's ability to distinguish a novel object from familiar objects after learning. Genetic inactivation of PP1 allowed shorter spacing to generate a significantly enhanced memory for objects, and this was correlated with better activation of CREB-dependent transcription [42]. It is not clear if spaced and massed training regulate PP1 activity differentially.

Another protein upstream of CREB is mitogen-activated protein kinase (MAPK) [61, 62], which is differentially activated after spaced and massed training in a number of systems [63, 64]. In cultured hippocampal neurons, four spaced 3 min depolarizations with 10 min rest periods evoke persistent activation of MAPK, whereas massing the depolarizations into one 12 min pulse failed to persistently activate MAPK [64]. In *Aplysia*, MAPK is required for synaptic facilitation [65]. As mentioned above, long-term sensitization in *Aplysia* can be formed by 4 or 5 spaced tail shocks (rest interval 15 min) [5]. However, 2 tail shocks spaced by 45 min can also lead to long-term sensitization, while 15 min and 60 min spacing were ineffective [63]. This narrow window for memory formation corresponds to a similar window of transient MAPK activity [63]. This transient window of MAPK activity was induced by the first tail shock, but was not sufficient to form long-term sensitization. Activation of MAPK following the first tail shock may provide a molecular mechanism allowing for the second tail shock applied in a specific temporal window to generate long-term memory [63]. These results suggest that MAPK activity plays an important role in defining the spacing intervals necessary for long-term memory formation. Recently, it was postulated that it is the conjunction of the activation of MAPK and that of the cyclic-AMP-dependent Protein kinase A (PKA) that was critical for induction of CREB and LTF [66]. Indeed, altering the spacing intervals to maximize overlap between activation of MAPK and that of PKA increased activation of CREB and the duration and magnitude of both LTF and long-term sensitization [66].

 While these studies show the importance of MAPK, they do not define how timing regulates MAPK. Insight into this question came from studies in *Drosophila* where the protein tyrosine phosphatase corkscrew (CSW), which is important for MAPK activation, was shown to directly define the intertrial interval necessary for long-term memory formation [67]. In *Drosophila*, the vertical lobes of the mushroom body neurons are required for long-term memory expression [68]. Overexpression of wild-type CSW in the mushroom body neurons of *Drosophila* during the odor avoidance task shortened the intertrial intervals necessary to induce long-term memory [67]. Furthermore, overexpression of constitutively active CSW proteins prolongs these resting intervals [67]. Interestingly, training that gives rise to long-term memory generates waves of MAPK activity dependent on corkscrew [67], further implicating corkscrew activity as a neural correlate for the spacing effect.

## 5. The Atypical Persistently Active PKC: A Downstrean Target of CREB?

What are the CREB-regulated target genes necessary for long-term memory formation after spaced training? A possible downstream target of transcription is PKM*ζ* which is a truncated, persistently active form of the atypical PKC*ζ*. PKM*ζ* was shown to be critical for the maintenance of long-term memories in different model organisms [56, 69, 70]. *Drosophila* bearing heat shock inducible mouse atypical PKM*ζ* (MaPKM*ζ*) could form enhanced memories in the odor-avoidance task after massed training, resembling those formed from spaced training [70]. However, timing of the activation of MaPKM*ζ* was critical for the memory enhancement: MaPKM*ζ* had to be induced 30 min after training since inducing it before training or 2 hours after resulted in no effect. Furthermore, inducing *Drosophila* atypical PKM (DaPKM) also enhanced long-term memory after massed training [70]. Moreover, inhibiting DaPKM activity resulted in the inhibition of long-term memory formation from spaced training, but not learning or short-term memory [70]. PKMs were also shown to play a critical role in long-term memory maintenance in *Aplysia* [69]. Cai and colleagues showed that intrahemocoel injections of the pseudosubstrate inhibitory peptide ZIP or the PKC inhibitor chelerythrine erased the memory for long-term sensitization (LTS) of the siphon-withdrawal reflex after spaced training as late as 7 days after training [69]. Furthermore, both PKM inhibitors disrupted the maintenance of LTF at the sensorimotor synapse [69]. In rodents, L-LTP maintenance is reversed by inhibiting PKM*ζ*, even when inhibitors are applied from hours to one day after LTP induction [71–75]. Moreover, several forms of long-term memory are rapidly erased by locally inhibiting PKM*ζ* in different brain regions of rats and mice, from days to even weeks and months after training [72, 73, 76–79]. Interestingly, an increase in PKM*ζ* levels was observed during the maintenance of LTP, and this increase was not due to proteolytic cleavage of PKC*ζ* but rather to de novo synthesis of PKM*ζ* from its own mRNA [80, 81]. A missing step in this story would be demonstrating that the mRNA for PKM*ζ* is regulated by CREB, and that increased PKM*ζ* levels are seen after spaced, but not massed training.

## 6. Differences between Spaced and Massed Training

Spaced and massed training generate different forms of synaptic plasticity by activating different signaling pathways. Our recent studies suggest that protein translation is regulated in a training-dependent manner and might represent one mechanism by which neurons distinguish between spaced and massed training. These studies will be discussed below.

 PKA and PKC are key proteins involved in memory formation. During sensitization in *Aplysia*, 5HT acts through G-protein coupled receptors to activate PKA and PKC [82–84]. LTF produced by spaced applications of 5HT depends on persistent activation of PKA [85, 86]; whereas massed applications of 5HT lead to both activation of PKA and of the calcium-independent PKC Apl II [85, 87, 88]. PKC activation is regulated in a training-dependent manner. Indeed, PKC translocation to the plasma membrane desensitizes less during massed training [87]. Since activation of PKC can inhibit PKA-mediated actions of 5HT [89], the suppression of PKC activity and the subsequent decrease in PKA inhibition is one mechanism through which spaced training might lead to long-term memory formation. Interestingly, this training-dependent regulation of PKC is mediated by competing feedback mechanisms that act through protein synthesis. Inhibiting protein translation using anisomycin inhibited desensitization of PKC Apl II translocation during spaced training but had the opposite effect on massed training where desensitization of PKC Apl II translocation was dramatically increased [87]. We hypothesized that during massed training, PKC would trigger synthesis of an antidesensitization protein (AD) that protects PKC Apl II translocation from desensitizing. During spaced training, PKA would trigger synthesis of a desensitization protein (D) that increases desensitization of PKC Apl II translocation [87]. We further developed a mathematical model of PKC translocation, which found that the rates of protein synthesis and degradation might play a role in its desensitization [19]. The model consisted of a system of integrodifferential equations describing the differential desensitization of PKC Apl II activation during massed and spaced training. The model provided predictions about the molecular mechanisms responsible for the differences between massed and spaced training, and these predictions were validated experimentally. In particular, the model successfully predicted changes in PKC translocation with different spacing paradigms [19]. The model suggested that the rates of protein synthesis and degradation were critical in determining the effects of spacing [19]. Specifically, our results suggested that increased desensitization during spaced applications of 5HT was due to the short half-life of the hypothetical protein, which prevented homologous desensitization (AD). One pulse of 5HT would synthesize this protein that would protect against desensitization for the short period before it was degraded. Massed application of 5HT would constantly replenish this protein, while spaced applications of 5HT, with an interapplication interval longer than the half-life of the protein, would overshoot the protective period and cause increased desensitization [19]. Another computational study found that the switch to persistent PKA activity after 4 pulses of 5HT, but not 3, was due to the zero-order ultrasensitivity of MAPK phosphorylation. It will be interesting to determine if these effects on MAPK activity are also downstream of synthesis of short-lived proteins. Protein synthesis and degradation rates are appealing mechanisms for sensing time in the range of spacing intervals (5–15 minutes in most cases). The levels of plasticity related proteins could increase in this time range [90, 91]. Regulation of ATF4, the CREB repressor, is a specific example of a protein with a short half-life that regulates memory [58, 59]. In this case, learning leads to decreased translation of ATF4 coupled to its fast degradation [59].

## 7. Conclusion

In summary, we reviewed the current knowledge supporting CREB as a key molecule involved in regulating the spacing effect. This function of CREB seems to be highly conserved throughout evolution. We further examined proteins upstream of CREB as well as the atypical persistently active PKC as a possible downstream target of CREB. Spaced training is reflected in differential CREB activation. The mechanism underlying this is not clear, but could be due to decreased translation of the CREB repressor, decreased levels of CREB phosphatase, differential activation of MAP kinase, or decreased activation of PKC. Moreover, based on our recent modeling studies, we suggest rapid degradation of newly synthesized proteins as a mechanism by which neurons distinguish between spaced and massed training in the time domain of minutes. An important challenge remains to identify the proteins which synthesis is regulated in this fashion in a training-dependent manner and how they interact with CREB activation.

## References

[1] Commins S, Cunningham L, Harvey D, Walsh D (2003). Massed but not spaced training impairs spatial memory. *Behavioural Brain Research*.

[2] Isabel G, Pascual A, Preat T (2004). Exclusive consolidated memory phases in *Drosophila*. *Science*.

[3] Josselyn SA, Shi C, Carlezon WA, Neve RL, Nestler EJ, Davis M (2001). Long-term memory is facilitated by cAMP response element-binding protein overexpression in the amygdala. *Journal of Neuroscience*.

[4] Margulies C, Tully T, Dubnau J (2005). Deconstructing memory in *Drosophila*. *Current Biology*.

[5] Sutton MA, Ide J, Masters SE, Carew TJ (2002). Interaction between amount and pattern of training in the induction of intermediate- and long-term memory for sensitization in *Aplysia*. *Learning and Memory*.

[6] Tully T, Preat T, Boynton SC, Del Vecchio M (1994). Genetic dissection of consolidated memory in Drosophila. *Cell*.

[7] Ebbinghaus H (1885). **Ű*ber das Gedächtnis: Untersuchen zur experimentallen Psychologie*.

[8] Goverover Y, Arango-Lasprilla JC, Hillary FG, Chiaravalloti N, Deluca J (2009). Application of the spacing effect to improve learning and memory for functional tasks in traumatic brain injury: a pilot study. *American Journal of Occupational Therapy*.

[9] Goverover Y, Hillary FG, Chiaravalloti N, Arango-Lasprilla JC, Deluca J (2009). A functional application of the spacing effect to improve learning and memory in persons with multiple sclerosis. *Journal of Clinical and Experimental Neuropsychology*.

[10] Kelley PA (2007). *Making Minds: What's Wrong with Education- and What Should We Do About It?*.

[11] Cepeda NJ, Pashler H, Vul E, Wixted JT, Rohrer D (2006). Distributed practice in verbal recall tasks: a review and quantitative synthesis. *Psychological Bulletin*.

[12] Vlach HA, Sandhofer CM, Kornell N (2008). The spacing effect in children’s memory and category induction. *Cognition*.

[13] Caporale N, Dan Y (2008). Spike timing-dependent plasticity: a Hebbian learning rule. *Annual Review of Neuroscience*.

[14] Kessels HW, Malinow R (2009). Synaptic AMPA receptor plasticity and behavior. *Neuron*.

[15] Malenka RC (2003). Synaptic plasticity and AMPA receptor trafficking. *Annals of the New York Academy of Sciences*.

[16] Yovell Y, Abrams TW (1992). Temporal asymmetry in activation of *Aplysia* adenylyl cyclase by calcium and transmitter may explain temporal requirements of conditioning. *Proceedings of the National Academy of Sciences of the United States of America*.

[17] Alberini CM (1999). Genes to remember. *Journal of Experimental Biology*.

[18] Frank DA, Greenberg ME (1994). CREB: a mediator of long-term memory from mollusks to mammals. *Cell*.

[19] Naqib F, Farah CA, Pack CC, Sossin WS (2011). The rates of protein synthesis and degradation account for the differential response of neurons to spaced and massed training protocols. *PLoS Computational Biology*.

[20] Frey U, Huang YY, Kandel ER (1993). Effects of cAMP simulate a late stage of LTP in hippocampal CA1 neurons. *Science*.

[21] Grecksch G, Matthies H (1980). Two sensitive periods for the amnesic effect of anisomycin. *Pharmacology Biochemistry and Behavior*.

[22] Huang YY, Li XC, Kandel ER (1994). cAMP Contributes to mossy fiber LTP by initiating both a covalently mediated early phase and macromolecular synthesis-dependent late phase. *Cell*.

[23] Kandel ER (2001). The molecular biology of memory storage: a dialogue between genes and synapses. *Science*.

[24] Montarolo PG, Goelet P, Castellucci VF (1986). A critical period for macromolecular synthesis in long-term heterosynaptic facilitation in *Aplysia*. *Science*.

[25] Nguyen PV, Abel T, Kandel ER (1994). Requirement of a critical period of transcription for induction of a late phase of LTP. *Science*.

[26] Carew TJ, Pinsker HM, Kandel ER (1972). Long-term habilitation of a defensive withdrawal reflex in *Aplysia*. *Science*.

[27] Gover TD, Abrams TW (2009). Insights into a molecular switch that gates sensory neuron synapses during habituation in *Aplysia*. *Neurobiology of Learning and Memory*.

[28] Glanzman DL, Mackey SL, Hawkins RD, Dyke AM, Lloyd PE, Kandel ER (1989). Depletion of serotonin in the nervous system of *Aplysia* reduces the behavioral enhancement of gill withdrawal as well as the heterosynaptic facilitation produced by tail shock. *Journal of Neuroscience*.

[29] Marinesco S, Kolkman KE, Carew TJ (2004). Serotonergic modulation in *Aplysia*. I. Distributed serotonergic network persistently activated by sensitizing stimuli. *Journal of Neurophysiology*.

[30] Mauelshagen J, Sherff CM, Carew TJ (1998). Differential induction of long-term synaptic facilitation by spaced and massed applications of serotonin at sensory neuron synapses of *Aplysia* californica. *Learning and Memory*.

[31] Alberini CM, Ghirardi M, Metz R, Kandel ER (1994). C/EBP is an immediate-early gene required for the consolidation of long-term facilitation in *Aplysia*. *Cell*.

[32] Bartsch D, Ghirardi M, Skehel PA (1995). *Aplysia* CREB2 represses long-term facilitation: relief of repression converts transient facilitation into long-term functional and structural change. *Cell*.

[33] Dash PK, Hochner B, Kandel ER (1990). Injection of the cAMP-responsive element into the nucleus of *Aplysia* sensory neurons blocks long-term facilitation. *Nature*.

[34] Kaang BK, Kandel ER, Grant SGN (1993). Activation of cAMP-responsive genes by stimuli that produce long-term facilitation in *Aplysia* sensory neurons. *Neuron*.

[35] Sossin WS (1996). Mechanisms for the generation of synapse specificity in long-term memory: the implications of a requirement for transcription. *Trends in Neurosciences*.

[36] Beck CDO, Schroeder B, Davis RL (2000). Learning performance of normal and mutant drosophila after repeated conditioning trials with discrete stimuli. *Journal of Neuroscience*.

[37] Ho IS, Hannan F, Guo HF, Hakker I, Zhong Y (2007). Distinct functional domains of neurofibromatosis type 1 regulate immediate versus long-term memory formation. *Journal of Neuroscience*.

[38] Yin JCP, Wallach JS, Del Vecchio M (1994). Induction of a dominant negative CREB transgene specifically blocks long- term memory in *Drosophila*. *Cell*.

[39] Akalal DBG, Yu D, Davis RL (2010). A late-phase, long-term memory trace forms in the *γ* neurons of *Drosophila* mushroom bodies after olfactory classical conditioning. *Journal of Neuroscience*.

[40] Yu D, Akalal DBG, Davis RL (2006). *Drosophilaα*/*β* mushroom body neurons form a branch-specific, long-term cellular memory trace after spaced olfactory conditioning. *Neuron*.

[41] Scharf MT, Woo NH, Matthew Lattal K, Young JZ, Nguyen PV, Abel T (2002). Protein synthesis is required for the enhancement of long-term potentiation and long-term memory by spaced training. *Journal of Neurophysiology*.

[42] Genoux D, Haditsch U, Knobloch M, Michalon A, Storm D, Mansuy IM (2002). Protein phosphatase 1 is a molecular constraint on learning and memory. *Nature*.

[43] Hummler E, Cole TJ, Blendy JA (1994). Targeted mutation of the CREB gene: compensation within the CREB/ATF family of transcription factors. *Proceedings of the National Academy of Sciences of the United States of America*.

[44] Bourtchuladze R, Frenguelli B, Blendy J, Cioffi D, Schutz G, Silva AJ (1994). Deficient long-term memory in mice with a targeted mutation of the cAMP- responsive element-binding protein. *Cell*.

[45] Kogan JH, Frankland PW, Blendy JA (1997). Spaced training induces normal long-term memory in CREB mutant mice. *Current Biology*.

[46] Blendy JA, Schmid W, Kiessling M, Schutz G, Gass P (1995). Effects of kainic acid induced seizures on immediate early gene expression in mice with a targeted mutation of the CREB gene. *Brain Research*.

[47] Maldonado R, Blendy JA, Tzavara E (1996). Reduction of morphine abstinence in mice with a mutation in the gene encoding CREB. *Science*.

[48] Balschun D, Wolfer DP, Gass P (2003). Does cAMP response element-binding protein have a pivotal role in hippocampal synaptic plasticity and hippocampus-dependent memory?. *Journal of Neuroscience*.

[49] Ilin Y, Richter-Levin G (2009). ERK2 and CREB activation in the amygdala when an event is remembered as “fearful” and not when it is remembered as "instructive". *Journal of Neuroscience Research*.

[50] Josselyn SA, Kida S, Silva AJ (2004). Inducible repression of CREB function disrupts amygdala-dependent memory. *Neurobiology of Learning and Memory*.

[51] Zhou Y, Won J, Karlsson MG (2009). CREB regulates excitability and the allocation of memory to subsets of neurons in the amygdala. *Nature Neuroscience*.

[52] Abel T, Nguyen PV, Barad M, Deuel TAS, Kandel ER, Bourtchouladze R (1997). Genetic demonstration of a role for PKA in the late phase of LTP and in hippocampus-based long-term memory. *Cell*.

[53] Bliss TVP, Collingridge GL (1993). A synaptic model of memory: long-term potentiation in the hippocampus. *Nature*.

[54] Martin SJ, Grimwood PD, Morris RGM (2000). Synaptic plasticity and memory: an evaluation of the hypothesis. *Annual Review of Neuroscience*.

[55] Matthies H, Reymann KG (1993). Protein kinase A inhibitors prevent the maintenance of hippocampal long-term potentiation. *NeuroReport*.

[56] Sacktor TC (2011). How does PKM zeta maintain long-term memory?. *Nature Reviews Neuroscience*.

[57] Pittenger C, Huang YY, Paletzki RF (2002). Reversible inhibition of CREB/ATF transcription factors in region CA1 of the dorsal hippocampus disrupts hippocampus-dependent spatial memory. *Neuron*.

[58] Chen A, Muzzio IA, Malleret G (2003). Inducible enhancement of memory storage and synaptic plasticity in transgenic mice expressing an inhibitor of ATF4 (CREB-2) and C/EBP proteins. *Neuron*.

[59] Costa-Mattioli M, Gobert D, Stern E (2007). eIF2*α* phosphorylation bidirectionally regulates the switch from short- to long-term synaptic plasticity and memory. *Cell*.

[60] Hagiwara M, Alberts A, Brindle P (1992). Transcriptional attenuation following cAMP induction requires PP-1- mediated dephosphorylation of CREB. *Cell*.

[61] Ait-Si-Ali S, Carlisi D, Ramirez S (1999). Phosphorylation by p44 MAP Kinase/ERK1 stimulates CBP histone acetyl transferase activity in vitro. *Biochemical and Biophysical Research Communications*.

[62] Michael D, Martin KC, Seger R, Ning MM, Baston R, Kandel ER (1998). Repeated pulses of serotonin required for long-term facilitation activate mitogen-activated protein kinase in sensory neurons of *Aplysia*. *Proceedings of the National Academy of Sciences of the United States of America*.

[63] Philips GT, Tzvetkova EI, Carew TJ (2007). Transient mitogen-activated protein kinase activation is confined to a narrow temporal window required for the induction of two-trial long-term memory in *Aplysia*. *Journal of Neuroscience*.

[64] Wu GY, Deisseroth K, Tsien RW (2001). Spaced stimuli stabilize MAPK pathway activation and its effects on dendritic morphology. *Nature Neuroscience*.

[65] Martin KC, Michael D, Rose JC (1997). MAP kinase translocates into the nucleus of the presynaptic cell and is required for long-term facilitation in *Aplysia*. *Neuron*.

[66] Zhang Y, Liu R-Y, Heberton GA (2011). Computational design of enhanced learning protocols. *Nature Neuroscience*.

[67] Pagani MR, Oishi K, Gelb BD, Zhong Y (2009). The phosphatase SHP2 regulates the spacing effect for long-term memory induction. *Cell*.

[68] Pascual A, Préat T (2001). Localization of long-term memory within the *Drosophila mushroom* body. *Science*.

[69] Cai D, Pearce K, Chen S, Glanzman DL (2011). Protein kinase m maintains long-term sensitization and long-term facilitation in *Aplysia*. *Journal of Neuroscience*.

[70] Drier EA, Tello MK, Cowan M (2002). Memory enhancement and formation by atypical PKM activity in *Drosophila melanogaster*. *Nature Neuroscience*.

[71] Ling DSF, Benardo LS, Serrano PA (2002). Protein kinase M*ζ* is necessary and sufficient for LTP maintenance. *Nature Neuroscience*.

[72] Madroñal N, Gruart A, Sacktor TC, Delgado-García JM (2010). PKM*ζ* inhibition reverses learning-induced increases in hippocampal synaptic strength and memory during trace eyeblink conditioning. *PLoS ONE*.

[73] Pastalkova E, Serrano P, Pinkhasova D, Wallace E, Fenton AA, Sacktor TC (2006). Storage of spatial information by the maintenance mechanism of LTP. *Science*.

[74] Sajikumar S, Navakkode S, Sacktor TC, Frey JU (2005). Synaptic tagging and cross-tagging: the role of protein kinase M*ζ* in maintaining long-term potentiation but not long-term depression. *Journal of Neuroscience*.

[75] Serrano P, Yao Y, Sacktor TC (2005). Persistent phosphorylation by protein kinase M*ζ* maintains late-phase long-term potentiation. *Journal of Neuroscience*.

[76] Hardt O, Migues PV, Hastings M, Wong J, Nader K (2010). PKM*ζ* maintains 1-day- and 6-day-old long-term object location but not object identity memory in dorsal hippocampus. *Hippocampus*.

[77] Kwapis JL, Jarome TJ, Lonergan ME, Helmstetter FJ (2009). Protein kinase mzeta maintains fear memory in the amygdala but not in the hippocampus. *Behavioral Neuroscience*.

[78] Shema R, Sacktor TC, Dudai Y (2007). Rapid erasure of long-term memory associations in the cortex by an inhibitor of PKM*ζ*. *Science*.

[79] von Kraus LM, Sacktor TC, Francis JT (2010). Erasing sensorimotor memories via PKM*ζ* inhibition. *PLoS ONE*.

[80] Hernandez AI, Blace N, Crary JF (2003). Protein kinase M*ζ* synthesis from a brain mRNA encoding an independent protein kinase C*ζ* catalytic domain. Implications for the molecular mechanism of memory. *Journal of Biological Chemistry*.

[81] Osten P, Valsamis L, Harris A, Sacktor TC (1996). Protein synthesis-dependent formation of protein kinase M*ζ* in long-term potentiation. *Journal of Neuroscience*.

[82] Dumitriu B, Cohen JE, Wan Q, Negroiu AM, Abrams TW (2006). Serotonin receptor antagonists discriminate between PKA- and PKC-mediated plasticity in *Aplysia* sensory neurons. *Journal of Neurophysiology*.

[83] Lee JA, Jang DJ, Kaang BK (2009). Two major gate-keepers in the self-renewal of neural stem cells: Erk1/2 and PLC1 in FGFR signaling. *Molecular Brain*.

[84] Nagakura I, Dunn TW, Farah CA, Heppner A, Li FF, Sossin WS (2010). Regulation of protein kinase C Apl II by serotonin receptors in *Aplysia*. *Journal of Neurochemistry*.

[85] Müller U, Carew TJ (1998). Serotonin induces temporally and mechanistically distinct phases of persistent PKA activity in *Aplysia* sensory neurons. *Neuron*.

[86] Sutton MA, Carew TJ (2000). Parallel molecular pathways mediate expression of distinct forms of intermediate-term facilitation at tail sensory-motor synapses in *Aplysia*. *Neuron*.

[87] Farah CA, Weatherill D, Dunn TW, Sossin WS (2009). PKC differentially translocates during spaced and massed training in *Aplysia*. *Journal of Neuroscience*.

[88] Sossin WS (1997). An autonomous kinase generated during long-term facilitation in *Aplysia* is related to the Ca2^+^-lndependent protein kinase C Apl II. *Learning and Memory*.

[89] Sugita S, Baxter DA, Byrne JH (1997). Modulation of a cAMP/protein kinase A cascade by protein kinase C in sensory neurons of *Aplysia*. *Journal of Neuroscience*.

[90] Nadif Kasri N, Nakano-Kobayashi A, Van Aelst L (2011). Rapid synthesis of the X-linked mental retardation protein OPHN1 mediates mglur-dependent LTD through interaction with the endocytic machinery. *Neuron*.

[91] Villareal G, Li Q, Cai D, Glanzman D (2007). The role of rapid, local, postsynaptic protein synthesis in learning-related synaptic facilitation in *Aplysia*. *Current Biology*.

